# A rat model of picornavirus-induced airway infection and inflammation

**DOI:** 10.1186/1743-422X-6-122

**Published:** 2009-08-11

**Authors:** Louis A Rosenthal, Svetlana P Amineva, Renee J Szakaly, Robert F Lemanske, James E Gern, Ronald L Sorkness

**Affiliations:** 1 Department of Medicine, University of Wisconsin School of Medicine and Public Health, 600 Highland Avenue, Madison, WI 53792, USA; 2 Morris Institute for Respiratory Research, University of Wisconsin School of Medicine and Public Health, 600 Highland Avenue, Madison, WI 53792, USA; 3 Department of Pediatrics, University of Wisconsin School of Medicine and Public Health, 600 Highland Avenue, Madison, WI 53792, USA; 4 School of Pharmacy, University of Wisconsin-Madison, Madison, WI 53705, USA

## Abstract

**Background:**

Infection of the lower airways by rhinovirus, a member of the picornavirus family, is an important cause of wheezing illnesses in infants, and plays an important role in the pathogenesis of rhinovirus-induced asthma exacerbations. Given the absence of natural rhinovirus infections in rodents, we investigated whether an attenuated form of mengovirus, a picornavirus whose wild-type form causes systemic rather than respiratory infections in its natural rodent hosts, could induce airway infections in rats with inflammatory responses similar to those in human rhinovirus infections.

**Results:**

After inoculation with 10^7 ^plaque-forming units of attenuated mengovirus through an inhalation route, infectious mengovirus was consistently recovered on days 1 and 3 postinoculation from left lung homogenates (median Log_10 _plaque-forming units = 6.0 and 4.8, respectively) and right lung bronchoalveolar lavage fluid (median Log_10 _plaque-forming units = 5.8 and 4.0, respectively). Insufflation of attenuated mengovirus, but not vehicle or UV-inactivated virus, into the lungs of BN rats caused significant increases *(P *< 0.05) in lower airway neutrophils and lymphocytes in the bronchoalveolar lavage fluid and patchy peribronchiolar, perivascular, and alveolar cellular infiltrates in lung tissue sections. In addition, infection with attenuated mengovirus significantly increased (*P *< 0.05) lower airway levels of neutrophil chemoattractant CXCR2 ligands [cytokine-induced neutrophil chemoattractant-1 (CINC-1; CXCL1) and macrophage inflammatory protein-2 (MIP-2; CXCL2)] and monocyte chemoattractant protein-1 (MCP-1; CCL2) in comparison to inoculation with vehicle or UV-inactivated virus.

**Conclusion:**

Attenuated mengovirus caused a respiratory infection in rats with several days of viral shedding accompanied by a lower airway inflammatory response consisting of neutrophils and lymphocytes. These features suggest that mengovirus-induced airway infection in rodents could be a useful model to define mechanisms of rhinovirus-induced airway inflammation in humans.

## Background

Human rhinovirus (HRV) infections are the most frequent cause of common colds and virus-induced asthma exacerbations, and wheezing HRV infections in infancy are associated with an increased risk for the development of childhood asthma [1-3]. A central conundrum with regard to HRV, a member of the picornavirus family, is explaining how a virus that usually causes a self-limiting upper airway infection, a common cold, can induce asthma exacerbations and provoke persistent lower airway sequelae in susceptible children [4,5]. An important clue in addressing this issue is the substantial evidence that HRV can infect the lower airways [6-11]. HRV infection of lower airway epithelial cells induces the secretion of a variety of proinflammatory cytokines, chemokines, and mediators [4].

Neutrophils are the predominant inflammatory cell initially recruited to the airways during HRV infections [12,13], and clinical studies have demonstrated that there is a positive correlation between this inflammatory response and respiratory symptoms and airway dysfunction [14-17]. Although these relationships have been observed in a variety of clinical and experimental infection studies, the nature of this relationship is still enigmatic. It is possible that 1) neutrophilic inflammation causes respiratory symptoms, 2) neutrophils recruited to the airways in response to HRV infection have antiviral effects and contribute to resolution of the infection, or 3) neutrophilic inflammation is an epiphenomenon that does not significantly affect the course of the disease. Finally, perhaps the difference between a relatively uneventful cold and more severe HRV-induced airway sequelae resides in the balance between beneficial and detrimental effects of the neutrophilic inflammatory response.

Progress in understanding the relationship between HRV infection, inflammation, and respiratory symptoms has been significantly hampered by the absence of rodent-specific rhinoviruses. Recently, murine experimental models have been established using either minor group HRV in wild-type mice or major group HRV in mice that are transgenic for human intercellular adhesion molecule-1 (ICAM-1; CD54), the receptor for major group HRV [18,19]. While these models will be useful, a significant drawback to these models is that HRV replication is short-lived (≤ 24 h) in the mouse. In studying the relationship between viral replication, inflammation, and respiratory dysfunction, it would be advantageous to develop a model with viral replication lasting several days, as occurs during clinical or experimental infections with HRV.

Mengovirus is a picornavirus that naturally infects rodents [20], and the native virus causes systemic infections that resemble poliovirus infections, rather than HRV infections, of humans. The poly(C) tract in the distal region of the 5' untranslated region of the mengovirus genome is a critical virulence determinant that inhibits interferon responses [21-25]. A panel of attenuated mengovirus mutants with varying deletions of the poly(C) tract (wild-type mengovirus has a poly(C) tract length of 44) has been derived, including vMC_0_, which has no poly(C) tract [21-25]. In contrast to the systemic and often lethal infections caused by wild type mengovirus, intracerebral or intraperitoneal administration of vMC_0 _induces self-limited infections, and vMC_0 _also stimulates vigorous type I interferon responses [21-25]. Furthermore, attenuated mengoviruses replicate well in epithelial cells but poorly in macrophage lineage cells [25]. These features are similar to those of HRV infection [4], and led us to hypothesize that inoculation of rats with vMC_0_ via inhalation could produce infection limited to the respiratory tract, and could serve as a model for HRV infections in humans.

## Results

### Expression of infectious virus in the lungs after inhalation of attenuated mengovirus

**Figure 1:**
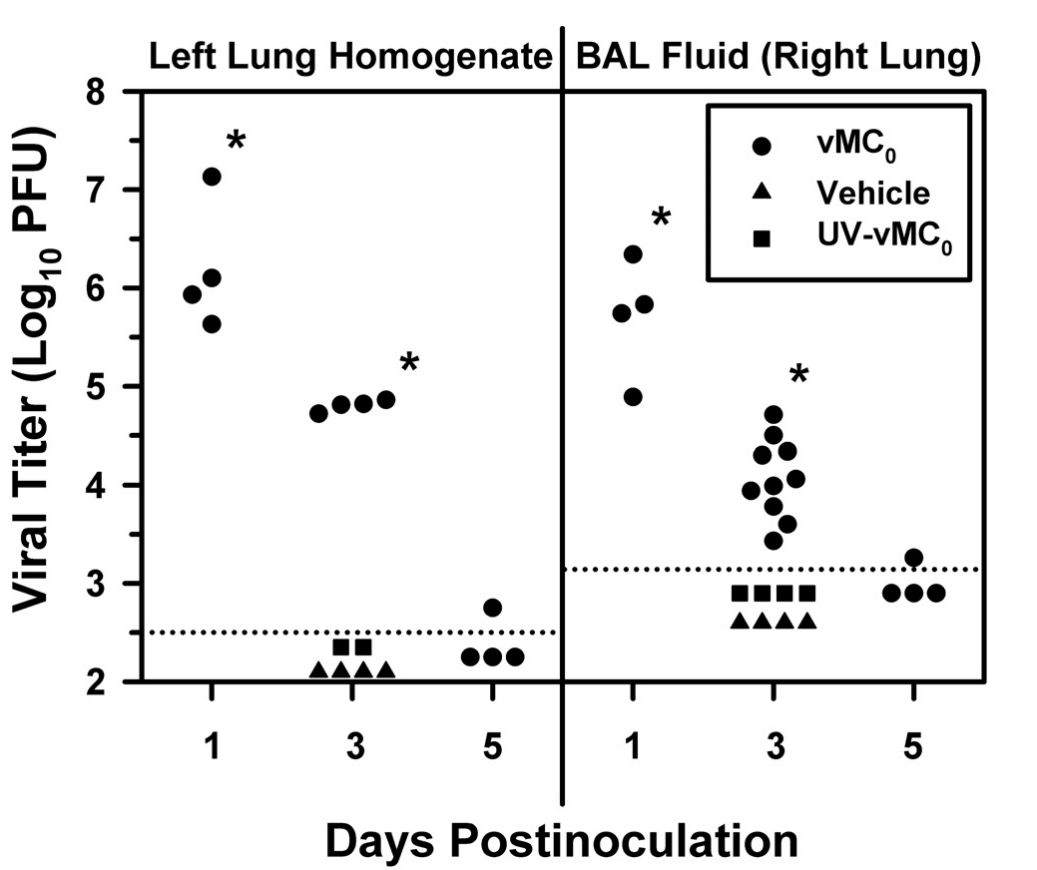
**Lung viral titers after inhalation of attenuated mengovirus**. Viral titers in left lung homogenates and BAL fluid (obtained from the right lung) from BN rats inoculated with 10^7 ^PFU of attenuated mengovirus, vMC_0_, an equivalent amount of UV-inactivated vMC_0_, or vehicle were determined by plaque assays. Data are the total amount of virus present in the lung homogenate or BAL fluid (virus concentrations were multiplied by the volumes of lung homogenate or BAL fluid). Symbols represent data from individual rats. Dotted lines indicate the limits of detection. * *P *< 0.005 (vMC_0 _vs. vehicle and UV-inactivated vMC_0_).

To examine whether attenuated mengovirus could induce lower airway infections in rats, 10^7 ^plaque-forming units (PFU) of attenuated mengovirus, vMC_0_, an equivalent amount of UV-inactivated vMC_0_, or vehicle were insufflated into the lungs of adult BN rats. On days 1 and 3 postinoculation, significant levels of infectious mengovirus were recovered from left lung homogenates (median Log_10 _PFU = 6.0 and 4.8, respectively) and right lung bronchoalveolar lavage (BAL) fluid (median Log_10 _PFU = 5.8 and 4.0, respectively) of BN rats inoculated with the attenuated mengovirus, vMC_0 _(Figure 1; *P *< 0.005). By day 5 postinoculation, viral titers in the lung homogenates and BAL fluid of vMC_0_-inoculated rats were either low or undetectable. Infectious mengovirus was not detected in lung homogenates and BAL fluid from BN rats inoculated with either UV-inactivated vMC_0 _or vehicle. Examination of brain, heart, and spleen homogenates and plasma revealed no evidence of systemic infection with vMC_0_.

### Reduction in body weight gain after inhalation of attenuated mengovirus

A reduction in body weight or in the rate of body weight gain is a sensitive measure of viral respiratory infections in rodents [26]. The percent gain in body weight from the day of the inoculation to day 3 postinoculation was significantly lower in BN rats inoculated with 10^7 ^PFU of vMC_0 _(median = 0.8%; n = 10 rats) than in those receiving the vehicle (median = 2.2%; n = 6 rats; *P *= 0.04). However, there was no significant difference between the percent gain in body weight in rats inoculated with UV-inactivated vMC_0 _(median = 1.6%; n = 5 rats) and those inoculated with vehicle, indicating the requirement for replication-competent virus for the observed effects on body weight.

### Development of neutrophilic lower airway inflammation after inhalation of attenuated mengovirus

**Figure 2:**
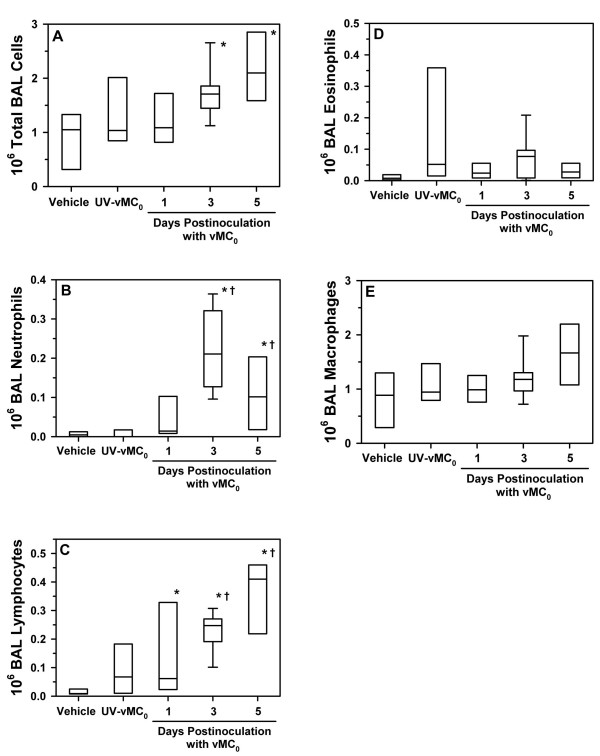
**Recruitment of neutrophils and lymphocytes to the lungs after inhalation of attenuated mengovirus**. Numbers of (A) total cells, (B) neutrophils, (C) lymphocytes, (D) eosinophils, and (E) macrophages in the BAL fluid harvested on days 1, 3, and 5 postinoculation from the right lungs of BN rats inoculated with 10^7 ^PFU of vMC_0 _(n = 4, 10, and 4 rats, respectively) and on day 3 postinoculation from those inoculated with an equivalent amount of UV-inactivated vMC_0 _(n = 5 rats) or vehicle (n = 7 rats). Data are presented as box plots. * *P *< 0.05 (mengovirus vs. vehicle); † *P *< 0.05 (vMC_0 _vs. UV-inactivated vMC_0_).

**Figure 3:**
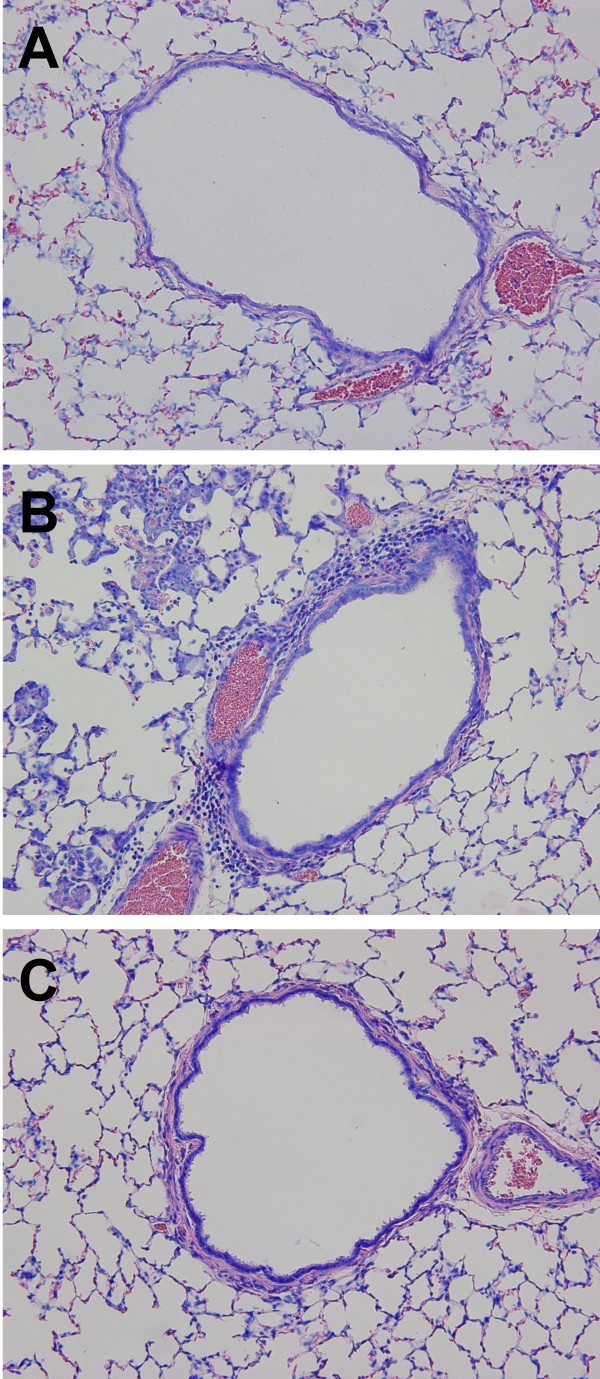
**Recruitment of inflammatory cell infiltrates to the lungs after inhalation of attenuated mengovirus**. Giemsa-stained sections of the left lungs from BN rats inoculated with (A) vehicle, (B) vMC_0 _(10^7 ^PFU) or (C) an equivalent amount of UV-inactivated vMC_0_. Lungs were harvested on day 3 postinoculation. Magnification, 20×.

Insufflation of vMC_0 _(10^7 ^PFU) into the lungs of adult BN rats induced the recruitment of neutrophils and lymphocytes into the lower airways. The total number of BAL cells and the numbers of BAL neutrophils and lymphocytes were significantly elevated on days 3 and 5 postinoculation in BN rats inoculated with attenuated mengovirus compared with those inoculated with an equivalent amount of UV-inactivated vMC_0 _or vehicle (Figure 2; *P *< 0.05). Levels of BAL lymphocytes were also significantly elevated on day 1 postinoculation in vMC_0_-inoculated BN rats as compared with vehicle-inoculated rats (Figure 2; *P *< 0.05). No significant differences were observed among the vMC_0_-, UV-inactivated vMC_0_-, and vehicle-inoculated groups with regard to the numbers of BAL macrophages or eosinophils. Examination of Giemsa-stained lung sections revealed patchy peribronchial, perivascular, and alveolar cellular infiltrates in the lungs of BN rats inoculated with 10^7 ^PFU of vMC_0 _but not in those inoculated with vehicle or UV-inactivated vMC_0 _(Figure 3). These data demonstrate the development of a neutrophilic and lymphocytic lower airway inflammatory response in rats after inhalation of attenuated mengovirus, which required replication-competent virus.

### Expression of CXCR2 ligands in the lower airways after inhalation of attenuated mengovirus

**Figure 4:**
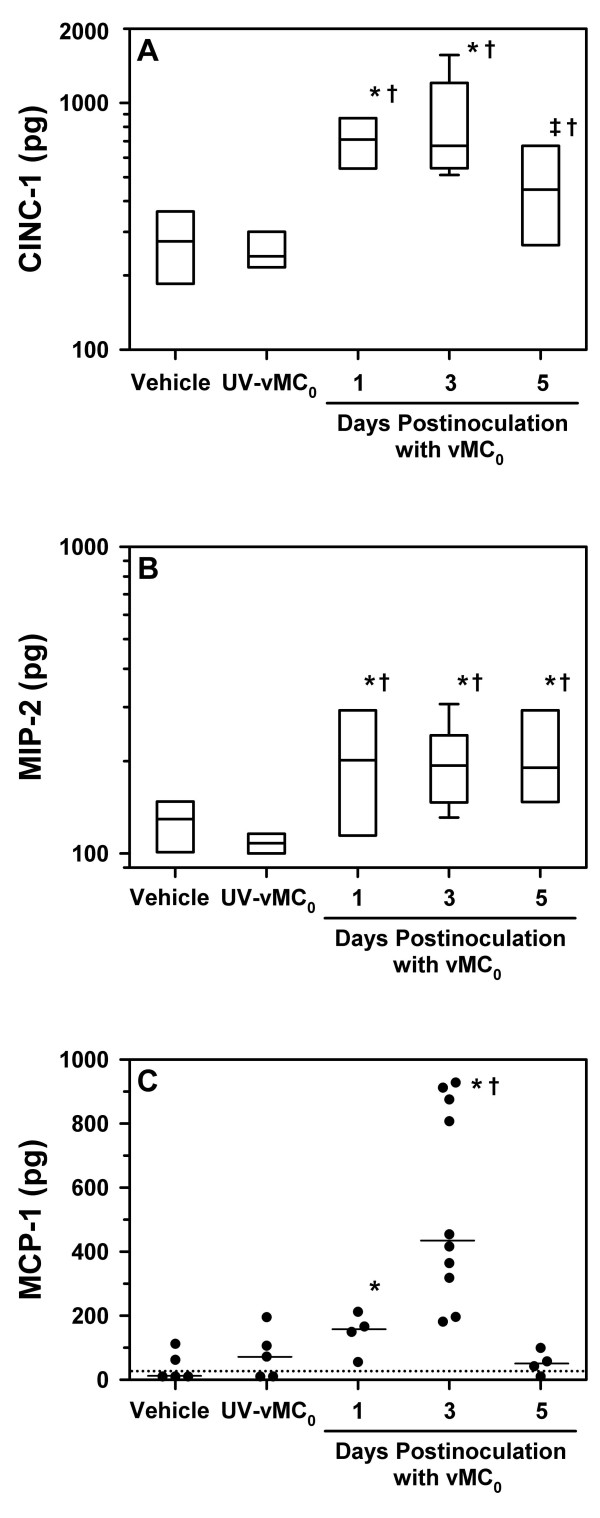
**Inhalation of attenuated mengovirus enhanced pulmonary expression of the chemokines, CINC-1, MIP-2, and MCP-1**. BAL fluid was harvested on days 1, 3, and 5 postinoculation from the right lungs of BN rats inoculated with 10^7 ^PFU of vMC_0 _(n = 4, 10, and 4 rats, respectively) and on day 3 postinoculation from those inoculated with an equivalent amount of UV-inactivated vMC_0 _(n = 5 rats) or vehicle (n = 5–6 rats), and (A) CINC-1, (B) MIP-2, and (C) MCP-1 levels were determined by ELISA. Data are the total amount of chemokine recovered from the right lung BAL (ELISA values, corrected for the 15× concentration, were multiplied by the BAL fluid volume). (A, B) Data are presented as box plots. (C) Symbols represent data from individual rats; bars indicate medians. * *P *< 0.05, ‡ *P *= 0.05 (vMC_0 _vs. vehicle); † *P *< 0.05 (vMC_0 _vs. UV-inactivated vMC_0_).

Given the significant neutrophilia in the lower airways that was induced in BN rats by inhalation of vMC_0_, we examined the BAL fluid for the expression of the rat CXCR2 ligands, CINC-1 and MIP-2, which are neutrophil chemoattractants [27]. The BAL fluid levels of CINC-1 and MIP-2 were significantly elevated on days 1, 3, and 5 postinoculation in rats inoculated with 10^7 ^PFU of vMC_0 _as compared with those inoculated with vehicle or an equivalent amount of UV-inactivated vMC_0 _(Figure 4A and 4B; *P *≤ 0.05).

### Expression of MCP-1 in the lower airways after inhalation of attenuated mengovirus

Because HRV infection induces high levels of MCP-1 expression [28], and MCP-1 indirectly contributes to neutrophil recruitment to the lungs [29-32], we examined the BAL fluid from BN rats that had been inoculated with 10^7 ^PFU of vMC_0 _for MCP-1 expression. The levels of MCP-1 in BAL fluid were significantly increased on days 1 and 3 or day 3 postinoculation in vMC_0_-inoculated rats compared with vehicle- or UV-inactivated vMC_0_-inoculated rats, respectively (Figure 4C; *P *< 0.05). As shown with regard to CXCR2 ligand expression, UV-inactivation of vMC_0 _abrogated its ability to induce a significant elevation in BAL fluid MCP-1 levels, demonstrating the need for replication-competent virus.

### Effect of inoculation dose on inflammatory response to inhalation of attenuated mengovirus

Inoculation with a ten-fold lower dose of vMC_0 _yielded a similar inflammatory response in the lower airways. Insufflation of 10^6 ^PFU of vMC_0 _into the lungs of BN rats (n = 4) induced a significant increase (*P *< 0.05) in the numbers [10^6 ^cells: median (interquartile range)] of neutrophils [0.19 (0.16, 0.21)] and lymphocytes [0.23 (0.20, 0.30)], but not total cells, eosinophils, or macrophages in the BAL fluid on day 3 postinoculation as compared with the values from vehicle-inoculated rats. In addition, the levels [pg: median (interquartile range)] of CINC-1 [715 (611, 835)], MIP-2 [188 (168, 208)], and MCP-1 [385 (266, 452)] in the BAL fluid were significantly elevated (*P *< 0.05) in these rats as compared with vehicle-inoculated controls. An inoculation dose of 10^5 ^PFU of vMC_0 _was substantially less effective at generating an inflammatory response in the lower airways of the rats, leading to the recruitment of about 75% fewer BAL neutrophils and 60% fewer BAL lymphocytes on day 3 postinoculation compared with that observed using inoculation doses of 10^7 ^or 10^6 ^PFU.

### Effect of inhalation of attenuated mengovirus on pulmonary physiology and airway hyperresponsiveness (AHR)

**Figure 5:**
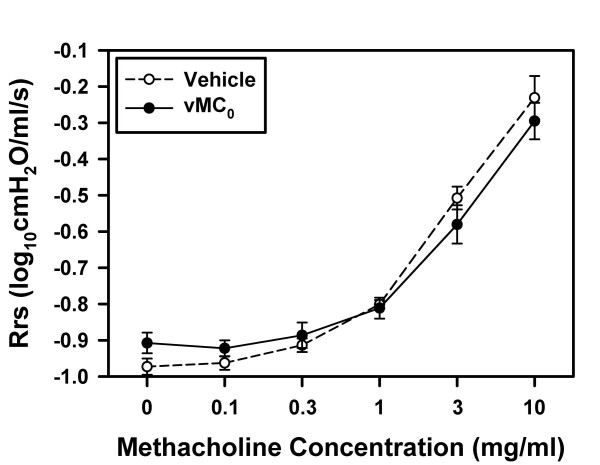
**Effect of inhalation of attenuated mengovirus on pulmonary physiology**. BN rats were inoculated with either vehicle or 10^7 ^PFU of vMC_0 _(n = 5 rats per group), and on day 3 postinoculation, pulmonary physiology measurements were obtained after exposure to aerosols of normal saline followed by escalating concentrations of methacholine. Values for respiratory system resistance (Rrs) are presented as the group means ± the standard error. There were no significant differences between the vehicle- and vMC_0_-inoculated groups.

To examine whether infection of the lower airways with attenuated mengovirus induced changes in pulmonary physiology, either vehicle or 10^7 ^PFU of vMC_0 _were insufflated into the lungs of adult BN rats, and pulmonary function was measured on day 3 postinoculation. No significant differences were observed between vehicle- and vMC_0_-inoculated groups of rats with regard to respiratory system resistance (Rrs) or the input impedance variables, Newtonian resistance (Rn), tissue viscance (G), and elastance (H), either at baseline or in response to methacholine challenge (Figure 5 and data not shown), indicating a lack of viral effects on pulmonary physiology and AHR.

## Discussion

The establishment of useful small animal models to study HRV pathogenesis has been an important goal to enable mechanistic studies and facilitate the development of new therapies. The earliest reported effort to develop a HRV infection model in mice required very large input doses of virus and pretreatment of the mice with actinomycin D [33]. Recently, more robust murine experimental models of HRV infection have been established. These models employ either a murine cell culture-adapted minor group HRV in wild-type mice or a major group HRV in mice that are transgenic for human ICAM-1 [18,19]. Although the development of these novel tools represents a significant advance in the study of HRV-induced airway inflammation, an important limitation is that HRV shedding is limited to ≤ 24 h postinoculation [18].

In the rat model described here, infectious mengovirus was consistently detected in the lungs at high levels, and persisted for at least 3 days after inoculation. The inoculation dose of 10^6^–10^7 ^PFU of attenuated mengovirus in the rats was similar to the dose of 5 × 10^6 ^TCID_50 _(50% tissue-culture infective dose) administered in the HRV models in mice [18,19], especially considering that the body weight of the rats is about an order of magnitude greater compared to that of mice. Furthermore, inhalation of attenuated mengovirus, but not vehicle or UV-inactivated virus, into the lungs of BN rats resulted in increases in chemokines (CINC-1, MIP-2, and MCP-1) and cellular inflammation (neutrophils, lymphocytes, and total BAL cells). Compared to the HRV mouse models, infection with vMC_0 _represents a rodent model of picornavirus-induced airway inflammation in which the roles of viral replication and persistence are more prominent.

Mengovirus-induced expression of CXCR2 ligands is consistent with the increased expression of CXCR2 ligands that is observed in response to rhinovirus infection [34-36]. A similar induction of CXCR2 ligands was also observed in the murine HRV infection models [18,19]. We also observed the induction of MCP-1 expression in response to inhalation of attenuated mengovirus, which represents another similarity between this rat model of attenuated mengovirus-induced airway inflammation and human host responses to HRV infection [28]. Therefore, the induction of rat CXC2 ligand and MCP-1 expression in airway fluids in response to inhalation of attenuated mengovirus closely resembles the HRV-induced enhancement of these chemokines.

Another similarity between this rat model and HRV infection in humans is the relative kinetics of the viral infection vs. the lower airway neutrophilic inflammatory response. Mengovirus titers in the lung peak earlier than the neutrophilic inflammatory response in the lower airways. This parallels data from experimental HRV inoculations in human volunteers [11,13]. In addition, the patchiness of the mengovirus-induced airway inflammation in this rat model is consistent with the patchy infection of airway epithelial cells observed in HRV infections in human subjects [11,37-39].

Infection of the lower airways with mengovirus did not result in significant changes in baseline pulmonary physiology measurements or in AHR to methacholine challenge in this rat model. It is important to note that experimentally naïve adult rats without existing airway disease were used in these studies. Similar to this rat model, several studies involving experimental HRV inoculations of healthy, nonasthmatic, nonallergic human subjects have demonstrated no changes in baseline pulmonary function or AHR after HRV infection [10,40-44]. In one study showing a small change in AHR after experimental HRV infection of nonasthmatic, nonallergic subjects, the small difference was only detected by employing a methacholine concentration that was a half-log higher than the highest concentration typically used [45]. In contrast, experimental inoculation with HRV has been shown to increase AHR in individuals with asthma and/or allergic rhinitis in several studies [10,17,44,46,47], although not in others [40,41,43,45,48]. Therefore, the absence of changes in AHR in these healthy adult rats without existing airway disease is consistent with the outcomes of experimental HRV infections in healthy humans who had no underlying airway disease, such as asthma or allergic rhinitis. The absence of viral effects on AHR in this mengovirus model and in the experimental HRV inoculations in humans is consistent with the murine experimental model of HRV infection described by Bartlett et al. in which there was no increase in AHR to methacholine challenge after HRV infection unless the BALB/c mice had also been sensitized and challenged with allergen [18]. However, in the murine experimental HRV infection model described by Newcomb et al., an increase in AHR to methacholine challenge was observed after infection of C57BL/6 mice with HRV [19], which may be related to the use of a different mouse strain. Overall, the lack of significant changes in pulmonary physiology during mengovirus-induced respiratory infection in adult rats without existing airway disease is consistent with previous observations in experimental HRV infections in humans. In future studies, it will be of interest to investigate the effects of mengovirus-induced respiratory infection on rats with existing airway injury related to prior exposures to allergens or other respiratory viruses [49] with the objective of modeling aspects of HRV-induced asthma exacerbations.

A potential limitation of this animal model is the use of mengovirus, which is neurotropic, to serve as a model for HRV, which primarily causes respiratory infections. In this regard, it is important to note that poliovirus, which is closely related to HRV, is also neurotropic. The attenuated mengovirus, vMC_0_, used in these studies induced a self-limited respiratory infection when administered through an inhalation route. This indicates that there is plasticity in the tissue tropism of vMC_0 _that makes it suitable for a model of picornavirus-induced airway infection and inflammation. Another consideration is that there are both similarities and differences in CXCR2 and its ligands between rats and humans [50]. Humans express IL-8 and two IL-8 receptors, CXCR1 and CXCR2, whereas rats do not express an IL-8 ortholog and only express CXCR2. However, rats do express relevant CXCR2 ligands, such as CINC-1 and MIP-2, which are functional analogs of IL-8 with regard to neutrophil recruitment and activation. We believe that the rat represents an attractive, relevant, and simplified model for examining the role of CXC chemokines in neutrophil recruitment and activation in response to picornavirus-induced respiratory infection because of the reduced number of chemokines and chemokine receptors to be examined.

## Conclusion

Overall, our data support the feasibility of using this novel rat model of picornavirus-induced lower airway infection and inflammation to study, among other questions, the role of neutrophilic inflammation in the host response to picornavirus-induced respiratory infections. Although this model does not fully encompass all aspects of HRV infection in humans, it does demonstrate a remarkable number of parallel developments that will provide novel opportunities to study the interactions between picornaviral replication and the host antiviral immune responses in a relevant small animal model.

## Methods

### Animals

BN/SsN male rats were purchased from Harlan (Indianapolis, IN) and had a median body weight of 250 g when used for inoculation studies. The rats were housed in HEPA-filtered isolation cubicles (Britz and Co., Wheatland, WY) in an American Association for Accreditation of Laboratory Animal Care-accredited laboratory animal facility at the University of Wisconsin School of Medicine and Public Health. All procedures were approved by the University of Wisconsin Animal Care and Use Committee and conformed to the Guide for the Care and Use of Laboratory Animals (1996).

### Virus

Stock preparations of the attenuated mengovirus, vMC_0 _(which has no poly(C) tract) [21-25], were prepared by transfection of HeLa cells with viral RNA transcribed from a plasmid encoding the vMC_0 _genome followed by amplification of viral titers via passage in HeLa cell cultures as described [51]. Supernates from uninfected HeLa cell cultures were used as vehicle controls, and UV-inactivated vMC_0 _stocks were prepared by exposing vMC_0 _to a germicidal UV lamp at a distance of 10 cm for 1 h. Plaque assays using HeLa cells were employed to determine the titer of the active virus preparations and to verify UV-inactivation. Active virus was undetectable (< 10 PFU/ml) in the UV-inactivated preparations.

### Virus inoculation

Rats were lightly anesthetized by inhalation of 5% isoflurane, and vMC_0_, UV-inactivated vMC_0_, or vehicle in a total volume of 0.1 ml were insufflated into the lungs via an orotracheal catheter.

### Measurements of pulmonary inflammation

At various times after inoculation, rats were anesthetized with urethane and euthanized by exsanguination. The chest was opened, and the left mainstem bronchus was clamped to allow BAL of the right lung. The right lung was filled with phosphate buffered saline (PBS) to total lung capacity by gravity and drained 5 times, the BAL fluid was centrifuged, and the cell pellet was resuspended in 1 ml PBS. The total number of BAL leukocytes was determined with an automated cell counter (model Z1, Beckman Coulter, Hialeah, FL), and cytospin slides were prepared for a differential leukocyte count based on 200 cells. BAL fluid was concentrated 15× using a centrifugal filter device with a molecular weight cutoff of 5,000 (Millipore, Bedford, MA) and stored at -80°C until analyzed for chemokine expression. Samples of unconcentrated BAL fluid were used for viral titer determinations. The left lung was either removed for viral titer determinations or filled to total lung capacity by gravity with 10% buffered formalin for histological analysis.

### Measurements of pulmonary physiology

Rats were anesthetized with pentobarbital (Abbott, North Chicago, IL), intubated via tracheostomy, paralyzed with succinylcholine HCl (Sigma, St. Louis, MO), and ventilated mechanically (flexiVent, SCIREQ, Montreal, Canada). Aerosol challenges were delivered by the ventilator via an inline nebulizer (Aeroneb, SCIREQ) as 10 breaths of aerosolized normal saline, followed by methacholine HCl (Sigma) solutions in concentrations of 0.1, 0.3, 1, 3, and 10 mg/ml. Each challenge was preceded by two lung inflations to 30 cmH2O, and the challenges were delivered every 4 min. After each aerosol challenge, measurements of pulmonary physiology were performed by the flexiVent system every 15 s for 2 min, alternating measures of Rrs with measures of input impedance variables (Rn, G, and H). For each variable, the highest value occurring after each aerosol challenge was recorded as the response, referenced to the value obtained after saline challenge.

### Measurement of viral titers

Viral titers in left lung homogenates, prepared in PBS (10% w/v) and clarified by centrifugation, and in unconcentrated BAL fluid were determined by plaque assay using HeLa cells as described [24,51]. Briefly, HeLa cell monolayers were inoculated with dilutions of the samples, incubated for 24–48 h at 37°C (until plaques form), formalin fixed, stained with crystal violet, and scored for plaques. Stock vMC_0 _preparations served as the positive control.

### Histological assessment of pulmonary inflammation

Sections (5 μM) were prepared from formalin-fixed, paraffin-embedded left lungs. Giemsa staining was performed on these sections, which were evaluated for inflammation by light microscopy.

### Measurement of chemokine expression

Chemokine levels in BAL fluid were determined using commercially available rat-specific enzyme-linked immunosorbent assay (ELISA) kits for CINC-1 (R&D Systems, Minneapolis, MN), MIP-2, and MCP-1 (Biosource, Camarillo, CA) with sensitivities of 7.8, 7.8, and 8 pg/ml, respectively, according to the manufacturers' instructions.

### Statistical analysis

Analysis of variance (general linear model) was performed on the BAL fluid CINC-1 and MIP-2 ELISA data and on pulmonary physiology data after a log transformation, and Fischer's least significant difference test was used for planned pairwise comparisons. A residual analysis was employed to test the adequacy of the models. Nonparametric tests were used to analyze all other data. For comparisons between two groups, the Mann-Whitney test was used. The Kruskal-Wallis test was used for comparisons among three or more groups and was followed by planned pairwise comparisons using the Mann-Whitney test. Because infectious virus was undetectable in the lung homogenate and BAL fluid samples from rats inoculated with vehicle or UV-inactivated virus, these groups were combined for statistical analysis of viral titers. Box plots depict the median and the interquartile range between the 25th and 75th percentile, and whiskers show the 10th and 90th percentiles. Analyses were performed using the statistical software package SYSTAT 11.0 (Systat Software, Chicago, IL).

## Competing interests

The authors declare that they have no competing interests.

## Authors' contributions

LAR co-conceived the study, designed and coordinated the experiments, participated in the animal and immunological studies, performed the data and statistical analysis, analyzed and interpreted the data, and drafted the manuscript. SPA carried out the virology studies and participated in the experimental design and interpretation of the data. RJS carried out the animal, immunological, and histological studies and participated in the interpretation of the data. RFL participated in the interpretation of the data and revision of the manuscript. JEG co-conceived the study and participated in the interpretation of the data and revision of the manuscript. RLS co-conceived the study and participated in the experimental design, the animal and immunological studies, the interpretation of the data, and the revision of the manuscript. All authors read and approved the final manuscript.
